# Shaping Modern Vaccines: Adjuvant Systems Using MicroCrystalline Tyrosine (MCT^®^)

**DOI:** 10.3389/fimmu.2020.594911

**Published:** 2020-11-24

**Authors:** Matthew D. Heath, Mona O. Mohsen, Pieter-Jan de Kam, Thalia L. Carreno Velazquez, Simon J. Hewings, Matthias F. Kramer, Thomas M. Kündig, Martin F. Bachmann, Murray A. Skinner

**Affiliations:** ^1^ Allergy Therapeutics (UK) Ltd, Worthing, United Kingdom; ^2^ Bencard Adjuvant Systems [a Division of Allergy Therapeutics (UK) Ltd], Worthing, United Kingdom; ^3^ Interim Translational Research Institute “iTRI”, National Center for Cancer Care and Research (NCCCR), Doha, Qatar; ^4^ Department of BioMedical Research, Immunology RIA, University of Bern, Bern, Switzerland; ^5^ Bencard Allergie (GmbH), München, Germany; ^6^ Dermatology, University Hospital Zurich, Zurich, Switzerland; ^7^ Jenner Institute, Nuffield Department of Medicine, University of Oxford, Oxford, United Kingdom

**Keywords:** adjuvants, virus-like particles, MicroCrystalline Tyrosine (MCT^®^), allergy, disease, immunization, Monophosphoryl Lipid A (MPL^®^), vaccines

## Abstract

The concept of adjuvants or adjuvant systems, used in vaccines, exploit evolutionary relationships associated with how the immune system may initially respond to a foreign antigen or pathogen, thus mimicking natural exposure. This is particularly relevant during the non-specific innate stage of the immune response; as such, the quality of this response may dictate specific adaptive responses and conferred memory/protection to that specific antigen or pathogen. Therefore, adjuvants may optimise this response in the most appropriate way for a specific disease. The most commonly used traditional adjuvants are aluminium salts; however, a biodegradable adjuvant, MCT^®^, was developed for application in the niche area of allergy immunotherapy (AIT), also in combination with a TLR-4 adjuvant—Monophosphoryl Lipid A (MPL^®^)—producing the first adjuvant system approach for AIT in the clinic. In the last decade, the use and effectiveness of MCT^®^ across a variety of disease models in the preclinical setting highlight it as a promising platform for adjuvant systems, to help overcome the challenges of modern vaccines. A consequence of bringing together, for the first time, a unified view of MCT^®^ mode-of-action from multiple experiments and adjuvant systems will help facilitate future rational design of vaccines while shaping their success.

## Introduction

### The Evolution of Vaccines and Adjuvants

The concept of variolation (human inoculation/insertion of pathogens) dates back to the 10^th^ century in China, here, immunization against small pox used the live virus itself. Edward Jenner practised variolation in the UK and moved the field to the next level in the last decade of the 18^th^ century by using a cowpox virus for immunization which eventually led to the first vaccine (derived from vaccinia virus from vacca, the Latin word for cow), and the eradication of small pox in the 20^th^ century (1–3). Again, during the early 1900’s the pioneering work of Louis Pasteur, Alexandre Yersin, and others was associated with the development of attenuated and inactivated vaccines which progressed for a variety of pathogens such as cholera, tetanus, polio, tuberculosis, and a severe pneumonia-form of plague (*Yersinia pestis*) (4–10).

“Discoveries [made] by accidents and sagacity, of things [the observers] were not in quest of” (1754, quoted in Merton and Barber 2004, p. 2) (11).

In the 1920’s, both Alexander Glenny and Gaston Ramon were working with diphtheria toxins (12, 13). Production of bacterial toxins became very efficient (14). It was not long before Glenny referred to the use of a toxoid in humans for the first time in 1923 (13). Serendipity has led to some of the greatest discoveries and breakthroughs in science and medicine over the past century. Indeed, the story of adjuvants begins with a French veterinarian who unlocked a secret weapon, at an intersection of chance and wisdom. Gaston Ramon’s (1886–1963) crucial discovery, whilst at the Pasteur Institute in Paris in the 1920’s, made the observation that “local infection” (or abscesses at the injection site) was in some way enhancing antibody (Ab) production (15). As such, a series of experiments were set out and by adding a variety of substances (e.g., agar and starch oil) to an inoculation—substances he referred to as adjuvants (from the Latin adiuvare, meaning to help or aid) - resulted in enhanced tetanus and diphtheria anti-serum production (15, 16). When Gaston Ramon discovered the immune potentiating effect of such adjuvants, the human population was reeling from the aftermath of the Spanish flu and faced burgeoning health risks from pathogens. Moreover, vaccination against viruses, for example, represented more of a challenge than vaccination against bacteria, mostly because it was more difficult to grow them.

As part of Glenny’s work dealing with bacterial toxins, metal salts (precipitates thereof) were employed during the purification process, the adsorbed toxoid was subject to the wisdom of Glenny to perform comparative immunological studies, which indicated greatly enhanced immunological effects (14, 17), illuminating the serendipitous points of discovery that have shaped the modern world. Today, optimized versions of “alum” salt precipitates [e.g., aluminium oxyhydroxide; AlO(OH), aluminium phosphate; AlPO4)] have been the mainstay of adjuvants in clinical vaccines for more than 70 years (16).

For most of this time, the scientific community considered the principle or “dogma” of explaining the effectiveness of aluminium adjuvants in the context of the “depot” effect - immune stimulation through prolonged exposure of the antigen (18, 19). However, more research devoted to this question has revealed evidence that better explains adjuvancy in the context of alums physicochemical attributes and biological properties than a depot effect alone (20–26).

Tools to study the genome or cellular systems have developed rapidly. This has inspired new strategies from empirical to rational approaches to vaccine design and antigen carrier (nano)-systems, for targeting both innate and adaptive immune responses in tackling more challenging or emerging diseases or improvements in safety and efficacy of others (27–29). Vaccines are disruptive technologies and one of the most cost-saving medical applications ever developed, and in the last decades, their application in non-infectious diseases such as allergy, cancer, diabetes, and even smoking cessation continue to be developed (30–34).

While recombinant vaccines have generally improved safety profiles compared with live-attenuated and whole-pathogen vaccines, they are also often less immunogenic due to the removal of their inherent pathogenic features and patterns. Modern vaccine development focusses on bridging or substituting this gap in order to improve their effectiveness without compromising safety. As a consequence, the development of new and sophisticated rational technologies such as antigen (nano)-carrier systems [e.g., virus-like particles (VLPs)] or combination of adjuvants (adjuvant systems) are being employed to help overcome these challenges (29, 35).

### Adjuvant Systems

Adjuvant Systems may comprise of a variety of classical adjuvants or immunomodulators that are combined and tailored for the specific antigen and target application. The immune system has evolved to recognise repetitive surface features like pathogen-associated molecular patterns (PAMPs), which forms the basic principles in how they are able to activate the innate immune system, which, in turn, leads to orchestration of a specific adaptive response.

The benefits of vaccines and immunization against pathogenic threats demonstrate a convincing positive benefit-risk ratio over many decades, with the scope to eradicate disease. The existing and evolving threats have been brought to light recently with the spread of SARS-Cov-2, which some have described as natures wake-up call to complacent civilisation; threatening our era of peak globalisation, which has grown under a safety net of medical and scientific advances. The consideration of adjuvants in new vaccine development can be the difference to what makes a vaccine effective or not. Particularly, so where pathogens with more complex life cycles with intracellular habits or pathogens with genetic variability exist. Optimizing vaccines for this purpose has been historically slow and cumbersome (e.g., influenza, HIV, and malaria) and often requires a more robust adaptive response. For billions of years, microbes have evolved in this way, and this complexity has only just begun to be better understood by scientists.

### Immunology, Immunization, and Immunotherapy

The innate and adaptive responses cover two broad phases of the body’s response to a pathogen or vaccine. Pattern recognition receptors (PRRs) on innate and adaptive immune cells [i.e., macrophages, dendritic cells (DCs), monocytes, neutrophils, and B cells] have evolved to recognise conserved features that are typical of pathogenic surface patterns [pathogen-associated molecular patterns (PAMPs)], thus being able to signal an incoming agent as a threat, that is distinguishable from “self” (16, 36, 37). PRRs will trigger intracellular signaling cascades, resulting in the production of pro-inflammatory cytokines. This early inflammatory response to infection or immunization is diverse and tightly regulated, its early orchestration shaping the quality in adaptive immunity. A key mediator in shaping the quality of this adaptive response are antigen-presenting cells (such as DCs, macrophages, and B cells), particularly where vaccines are concerned (16).

**Figure 1:**
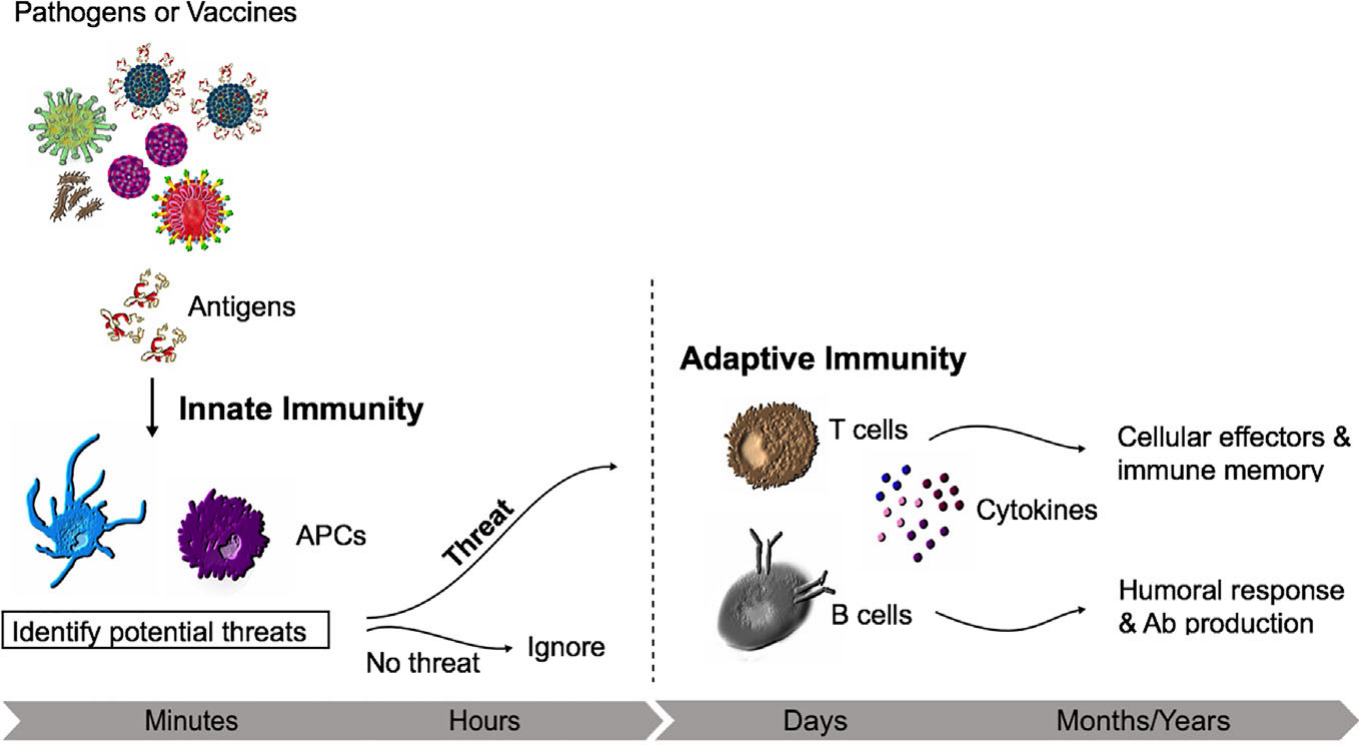
Innate and adaptive immunity time course. The non-specific early inflammatory response is characterized by cells of the innate immune system (e.g., Macrophages) which will recognise conserved repetitive features from bacteria or viruses. If recognized as a threat, the adaptive immune responses develops with the activation of lymphocytes.

How effectively a pathogen is removed will depend on the interplay between the innate and adaptive response and the quality that sits behind this immune reaction. In essence, the immune response to infection involves innate immune activation and antigen-specific responses of B and T cells, with the ideal vaccine typically able to induce Th1/Th17 immune responses that can direct this toward inactivation and removal of the threat, followed by development of immune memory (**Figure 1**) (16).

Allergy or parasitic infections are somewhat distinct, inducing strong type-2 immune responses. While Th2/IgE responses control parasitic infections, robust response to parasitic infections is also associated with allergic phenomena (38). Type 1 allergy is mediated by specific IgE, which results in an exaggerated immune response against an otherwise harmless substance. However, growing evidence of a negative association between parasitic infections and allergy at an ecological level highlights the complex inter-relationship between the two (39). Allergic disease is considered a new epidemic of the 21^st^ century, a burgeoning disease particularly in urban areas (40). The most effective way to treat IgE-mediated allergies is through allergen-specific immunotherapy (AIT), which entails repeated administration of specific allergens to patients resulting in protection against the allergic and inflammatory reactions (41). Despite its success, subcutaneous immunotherapy is generally slow and cumbersome for the patient. However, the advances in vaccinology may be exploited here too with the advent of new antigen nano-carriers, modified ways in presenting the allergen and next-generation adjuvants that may advance treatment for chronic diseases and emerging/re-emerging diseases into the modern world (42).

### Tailoring Adjuvant Systems

The combination of adjuvants (adjuvant systems) have been pioneered for the last few decades and has resulted in significant advancements in vaccine design and treatments. However, only few alternative adjuvants (other than alum) have been approved for human use (**Table 1**).

**Table 1:** Adjuvants used in licensed vaccines and immunotherapy [adapted from Di Pasquale et al. (16)].

Adjuvant	Composition	Immunomodulation	Product Indications
Aluminium (alum)	Aluminium salts mixed with antigens aluminium oxyhydroxide; AlO(OH), aluminium phosphate; AlPO4	Th2-biased, prolonged immune exposure (DC uptake), DAMP, Inflammasome activation, potent innate/Ab and inflammatory responses	Diphtheria, tetanus, pertussis, poliomyelitis, hepatitis A, hepatitis B, meningococcal, pneumococcal
Virosomes	Phospholipid membrane (either a mono- or bi-layer) vesicle incorporating virus derived proteins	Target antigen-presenting cells (APCs) and B cells	Hepatitis and influenza
AS03/ Oil-in-water/ MF59	Squalene-based	Increase antigen uptake by APCs, Ab B cell responses,	Influenza pandemic and seasonal.
AS04	Aluminium salt; AlO(OH) 3-deacyl-monophosphoryl lipid A	Increase antigen uptake by APCs, TLR-4 agonist, Th1 –biased Ab responses	Hepatitis B and Human Papillomavirus
AS01	Liposome-based 3-deacyl-monophosphoryl lipid A Purified saponin; QS-21	Th1-immunity Early innate inflammasome activation, Antigen-specific CD4+ T cells in addition to antigen-specific Abs, robust IFN-γ response.	Recombinant zoster vaccine (Shingrix, RZV). Mosquirix (*Plasmodium falciparum*; RTS,S’).
Montanide ISA51	Mineral oil	Increase antigen uptake by APCs, Ab B cell responses	Non-small cell lung cancer
MCT^®^	Crystalline form of L-Tyrosine (MicroCrystalline Tyrosine); MCT^®^	Biodegradable depot (43, 44), Th1-biased, Increase antigen uptake by APCs, highly immunogenic B and T cell responses (45).	Pollinex^®^ short-course allergy immunotherapy.
MCT^®^-MPL^®^	Crystalline form of L-Tyrosine (MicroCrystalline Tyrosine); MCT^®^ 3-deacyl-monophosphoryl lipid A	Th1-biased, Increase antigen uptake by APCs, highly immunogenic B and T cell responses. TLR-4 agonist, Th1 –biased Ab responses (46, 47),	Pollinex Quattro^®^ short-course allergy immunotherapy (48, 49).

Antigen carrier systems such as VLPs can be engineered to optimise antigen presentation and harbour intrinsic adjuvanticity, as these can be packaged with immunomodulators/adjuvants or combined with depot adjuvants to further tailor and optimise the immune response appropriately (29, 30, 50). The most commonly used traditional adjuvants are aluminium salts; however, for decades, a biodegradable adjuvant based on the crystalline form of the non-essential amino acid L-Tyrosine, MCT^®^, has been utilized in the niche area of allergy immunotherapy (43, 45). It is only in the last decade that its use and effectiveness across a variety of challenging disease models in the preclinical setting highlights it as a promising platform for adjuvant systems to help overcome the challenges associated with modern vaccines and challenging diseases (29).

The application of MCT^®^ as an adjuvant has more recently been extended across a broader vaccine scope with and without VLP antigen carrier systems; one such VLP system uses the cucumber mosaic VLP (CuMV_TT_), which includes intrinsic adjuvant features such as an engineered universal T helper cell epitope (CD4^+^, based on the tetanus toxin) and encapsulated RNA (TLR7/8 agonists) (51). The disease challenge models which have screened MCT^®^ -adjuvanted vaccines consist of largely murine data (malaria and cancer melanoma models), and one Ferret model (H1N1 Influenza) (50, 52–54). It is important to note that the proof of concept disease models capture biomarker measurements indicative of protection (efficacy) compared to control groups, with performance of the vaccine assessed against groups formulated with alum. Extended pharmacokinetic experiments featured in the Melanoma model (VLP-MCT^®^)provides unique insights into the importance of the depot effect of MCT^®^ when combined with nanoparticles in orchestrating a robust adaptive cytotoxic T cell response (50).

## MCT^®^ and Monophosphoryl Lipid-A in Allergy Immunotherapy

MCT^®^ is a biodegradable depot adjuvant developed primarily for use in short-course subcutaneous allergy immunotherapy (AIT), in combination with native allergens or modified allergens (allergoids) with or without Monophosphoryl Lipid A^®^ (MPL^®^, a Toll-like receptor 4 agonist) (48). Allergoid MCT^®^-MPL^®^ formulations are referred to as Pollinex Quattro^®^. Clinical evidence for the use of allergoid-MCT^®^-MPL^®^ adjuvant systems in allergy immunotherapy is well documented (55, 56). Combining an allergoid with an adjuvant system pays tribute to the short-course posology of the vaccine, which is administered in four to six injections within a year pre-seasonally, as opposed to longer-treatment courses (>30 injections) that are commonly applied in AIT and which are typically combined with alum depots (57).

The most recent phase II studies (including the optimal dose levels planned for Phase III) have recently been published for a six-injection presentation of Pollinex Quattro (PQ) Birch and PQ Grass (49, 55). These products are subject to further clinical development, and a phase III trial for both PQ Birch and PQ Grass are currently planned. Furthermore, a combined transcriptomic and proteomic biomarker analysis is pending in a phase III study for PQ Grass, while a smaller preliminary data set is available from an earlier trial, establishing some initial hypotheses related to mode-of-action/predictive efficacy biomarkers (46). Furthermore, Pollinex Quattro is listed in the current European Academy of Allergy and Clinical Immunology (EAACI) AIT guidelines with grade IA recommendation (56).

The PQ products employing the MCT^®^ and MPL^®^ adjuvant system are designed to desensitise allergic individuals by modulating the inherent Th1/Th2 imbalance of atopic disease. The mechanism involved in MCT^®^ -MPL^®^ adjuvancy has not been fully elucidated, but the synergistic attenuation of IgG may prolong protective immunity, which is a further benefited by combining the two adjuvants. The added benefit of MPL^®^ has been demonstrated in the clinic too (58). Several possible mechanisms might account for Toll-like receptor 4 (TLR-4) mediated effects in atopy and asthma. For instance, signaling through the TLRs is generally associated with production of Th1 cytokines by DCs *via* IL-12, leading to increased IFN-γ production (59).

For the PQ product portfolio, in total, 26 Phase I-III clinical trials have been conducted using various allergoids, with different formulations and dosing posologies, including 4695 patients in total (**Table 2**).

**Table 2:** Overview of clinical studies performed with Pollinex Quattro (PQ) products (Data on file, Allergy Therapeutics Plc).

	Phase I	Phase II	Phase III	Total
PQ Ragweed	1	3	1	5
PQ Grass	4	8	1	13
PQ Tree	1	3	1	5
PQ Birch	0	2	1	3

The combination of MCT^®^ and MPL^®^ has been shown to be safe and well tolerated in these Phase I-III studies and based on post-marketing data, i.e., >150,000 individuals have received PQ treatment (2004–2019) and an estimated >450,000 treatment courses have been dispensed (Data on file, Allergy Therapeutics plc). Moreover, the safety of MPL^®^ has been demonstrated in several products using MPL^®^ as an adjuvant (60). MPL^®^ is currently used as an adjuvant in the licensed product Cervarix (human papilloma virus vaccine), Fendrix (hepatitis B vaccine), and Shingrix [herpes zoster (shingles)] (60). Since first being licensed in 2006, over 200 million doses of HPV vaccines have been distributed globally, no significant safety issues have been observed (WHO, 2016).

In relation to MCT^®^, 1575 patients have received MCT^®^ alone as placebo group in placebo controlled GCP studies (including 9 million injections of all MCT^®^ platforms) (Data on file, Allergy Therapeutics plc). MCT^®^ as an adjuvant alone has been shown to be safe and well tolerated, without any treatment related serious adverse events (SAEs) being reported and no relevant effects observed in safety laboratory and vital signs. In a recent position paper, authored by an independent taskforce of EAACI members, a review of adjuvants and formulations currently used in marketed allergy immunotherapies discussed, stating, *“Since its introduction into AIT in 1970, there are no specific safety concerns known for MCT^®^. It can be anticipated that this fully biodegradable adjuvant will also in future studies not reveal side effects”* (61).

## MCT^®^ Mode of Action

In depth comparative adjuvant studies are, in general, limited in number, which may in part be due to the proprietary nature of investigational adjuvants. Since alum is the adjuvant of choice and most broadly studied, it is a useful comparator to use when studying vaccine mode-of-action.

MCT^®^ and alum have been compared head-to-head in a number of preclinical mouse models. In one such study, MCT^®^ combined with Ovalbumin stimulated striking and comparable B cell responses (antigen-specific IgG1, IgG2a, IgG2b, and IgG3) (45). The relevant induction of IgE was of interest, since IgE antibodies (Abs) are the key mediator of the allergic response and an “unwanted” reaction. Here, MCT^®^ triggered less IgE production than alum. This is an observation that has been consistently described in other studies, highlighting a key benefit in using a Th1-biased depot adjuvant in AIT and its reported synergy when combined with MPL^®^ as an adjuvant system (45, 52–54). The specific T cell (CD4+) cytokine response may, in part, explain this since MCT^®^ induced a more Th1-biased response. Of note, IL-4 is required for the Ig switch to IgE, and the lower propensity to induce IL-4, compared to alum, supports this notion (45). Both Alum and MCT^®^ were found to activate the inflammasome but this activation was not essential for the stimulation of B and T cell responses, nor early inflammatory markers (i.e., eosinophils and neutrophils) which were induced by MCT^®^ and alum adjuvants, when assessed by peritoneal lavage (45). Similar results have been reported for alum in mice deficient in IL-1R or NLRP3 (26, 62). Hence, although alum and MCT^®^ may activate the inflammasome *in vitro*, this does not affect the adaptive immune response needed for Ab production in AIT. Furthermore, increased B and T cell responses induced with alum or MCT^®^ -based vaccines did not depend on signaling through toll-like receptors, which is distinct from the TLR agonist MPL^®^ (45).

MCT^®^ ‘s half-life at the injection site was modelled in preclinical models, with an estimated half-life of 48 hours (44). MCT^®^ has a broad adsorption capacity with model allergens and carriers such as VLPs (63). The depot effect has been characterized with VLP nanoparticles, and this prolonged immune exposure was attributed to play an important role in priming T cells and, in particular, stimulating cytotoxic T cells—a response in which other adjuvants struggle to confer (50).

Shardlow and Exley have further characterized the physicochemical properties of MCT^®^, which describes needle-like crystalline structures, some of which stack together, to produce a high degree of structural order (64). The resultant crystals combined to form extensive rod-like features the majority of which exceeded 10 µm in length under physiological conditions (median size ca. 21 µm). MCT^®^ also appeared to lack a water decomposition phase by Thermogravimetric analysis, which indicated the lack of physically adsorbed moisture at the surface interface. A decrease in hydroxyl display/surface functionality has been associated with the reduced reactivity of aluminium salts *in vitro* in terms of proinflammatory cytokine production, reactive oxygen species (ROS) generation and inflammasome activation. The size of MCT^®^ may influence its recognition and uptake by THP-1 macrophages *in vitro* (64). In general, adjuvant particles between 1 and 3 µm in size have been considered optimal for recognition and engulfment by macrophages (65). The large hydrodynamic length of MCT^®^ crystals in biological medium (>ca. 10 µm) appeared to partially stymie the scavenging capacity of THP-1 macrophages *in vitro* (64). This may contribute to the safety profile of MCT^®^, since limited macrophage uptake may prohibit transport *via* barriers such as blood-brain and rapid transport to lymph nodes. The lower propensity to induce IgE/Th2-polarized responses and early inflammatory responses compared to alum, as described in Leuthard et al., 2018, may be partly attributed to the size and distribution of larger and more ordered crystalline structures of L-Tyrosine (45). This is in stark contrast to results obtained using a crystalline aluminium adjuvant where its optimal particle size (median size, 1.4 µm) appeared to more readily facilitate cytoplasmic loading (64).

**Figure 2:**
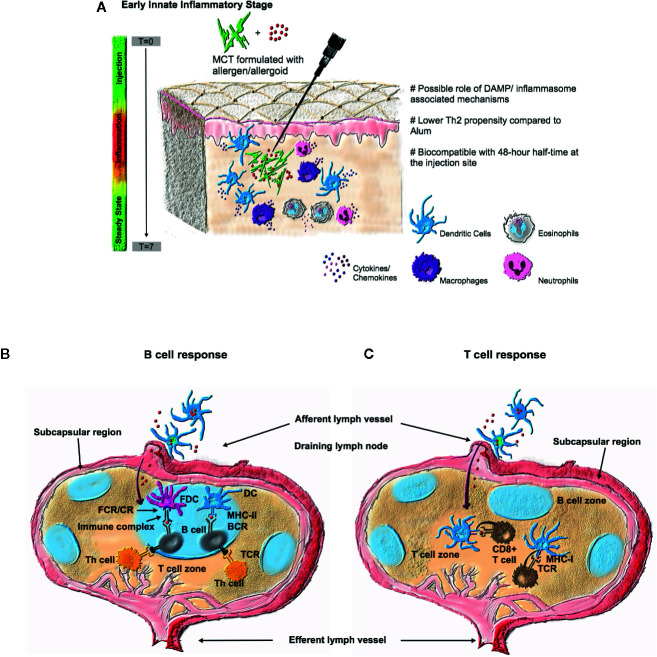
An overview of the immune response after vaccination with an MCT^®^ depot. **(A)** The early innate response is characterized by immediate exudation of neutrophils and eosinophils *in vivo*. The role of inflammasome/DAMP-associated mechanisms have not been precisely defined. The innate response has recorded an increase in dendritic cells (DCs), observed 24 h post-injection (45). MCT^®^ is biodegradable/biocompatible with an estimated half-life of 48 h at the injection site (44). As a result, it is cleared within 7 days with a return to a local steady state. The biodegradable depot properties of MCT^®^ are thought to be key in orchestrating the subsequent adaptive response**. (B)** The infiltrating antigen presenting cells to the draining lymph node, induce sustained and robust B cell response, *via* MHC class II antigen presentation (45, 52–44, 54), with sustained IgG antibody titers. The prolonged immune exposure of antigen is thought to further DC uptake and initiate CD4 T helper cell (Tfh) clonal expansion and differentiation (45). Furthermore, immune complexes may form with follicular dendritic cells (FDCs) *via* Fcγ receptors (Cd16 and CD32) and complement receptors (CD35). **(C)** The depot properties of MCT^®^ have been shown to be key in generating a more robust cytotoxic T cell response, thus the priming of T cells combined with optimal antigen delivery, such as when combined with VLPs, are key drivers in orchestrating this arm of the adaptive response (50).

Both adjuvants were characterized by immediate infiltration of neutrophils and eosinophils (MCT^®^ to a lesser degree) (45). This was the only study, to our knowledge, to characterize such inflammatory responses for MCT^®^. Although many innate reactions are important for the onset of adaptive immunity, the role of inflammasome activation in immunization and AIT has not been precisely defined. Indeed, MCT^®^ harbors different physicochemical properties, such as particle size, morphology, adsorption characteristics, and local pharmacokinetics compared to alum, which undoubtedly plays a pivotal role in shaping the quality of the Th1/Th2 biological response. MCT^®^’s roles in the innate and adaptive response are outlined in **Figure 2**.

### MCT^®^ -MPL^®^ “Synergy”

**Figure 3:**
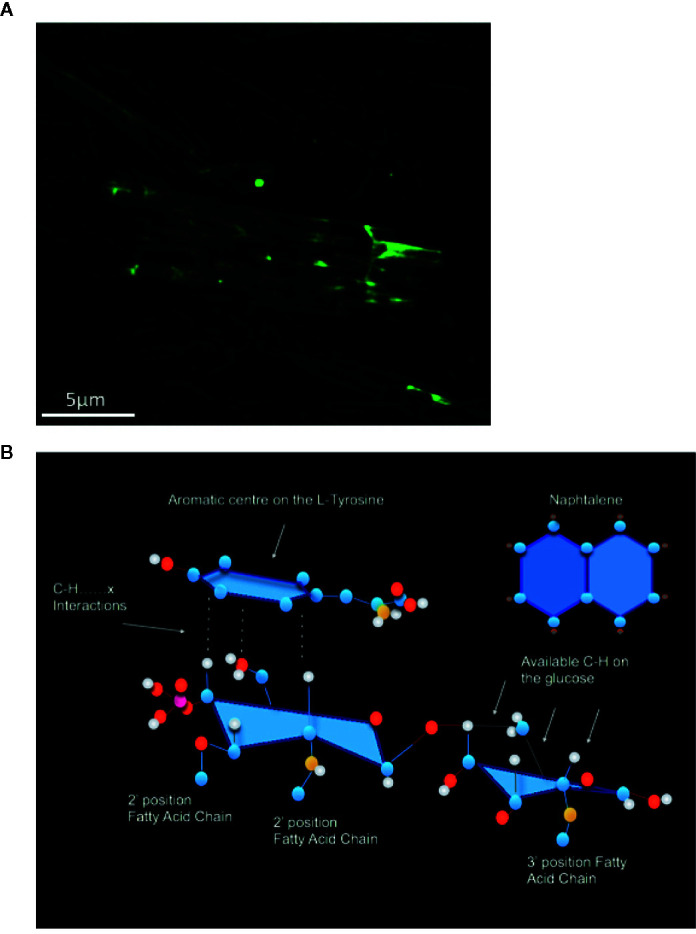
**(A)** The physical association of MPL^®^ across the needle-like crystalline structure of 20 mg/ml MCT^®^ has been characterized using fluorescently labeled LPS (100 µg; Lipopolysaccharide) as a substitute for MPL^®^ *via* confocal microscopy. **(B)** Proposed C–H⋯π interactions between the 2-deoxy-2-aminoglucose on MPL^®^ and the aromatic ring on L-tyrosine, based on inhibitor studies with Naphthalene (Adapted from Bell et al., 2015).

The physical association of MPL^®^ for MCT^®^ has been characterized using fluorescently labeled LPS (Lipopolysaccharide) as a substitute for MPL^®^ (**Figure 2**). The LPS was labeled with fluorescein isothiocyanate (FITC). Through confocal microscopy, it was possible to see that the labelled LPS is associated with the MCT^®^ depot. Furthermore, in Bell et al., 2015 allergoid and MPL^®^ adsorption to MCT^®^ in PQ allergy AIT formulations was determined *in vitro* using specific allergen IgE allergenicity and MPL^®^ content methods (63). The predominant mode (i.e., force) of adsorption between MPL^®^ and MCT^®^ was investigated by competition inhibitor binding experiments. This was predominantly inferred as C–H⋯π interactions between the 2-deoxy-2-aminoglucose backbone on MPL^®^ and aromatic ring of L-tyrosine in MCT^®^ (63) (**Figure 3B**). Furthermore, the physical association of MPL^®^ across the needle-like crystalline structure of 20 mg/ml MCT^®^ has been characterized using fluorescently labeled LPS as a substitute for MPL^®^ *via* confocal microscopy (**Figure 3A**).

Immunological synergy has been documented in allergoid formulations with or without MPL^®^, highlighting a synergistic relationship in IgG induction (47, 58). Formulation science is often an overlooked or under-appreciated discipline and often adjuvants may be included into formulations without having an extended level of characterisation of their interactions and compatibility with active substances and/or other adjuvants. Indeed, adsorption characteristics of adjuvants may shape bio-availability and in turn vaccine effectiveness (ratio of free versus adjuvants bound antigen may determine antigen draining kinetics) (28). MCT^®^ demonstrates consistent adsorption characteristics, when combined with antigens and allergoids (63). As such, quality attributes may be controlled for over the course of the products shelf life and investigated in preclinical immunogenicity models of the disease to further tailor and optimise properties of vaccines.

### MCT^®^ Combined With Poorly Immunogenic Antigens

It is important to note that the combination of MCT^®^ with poorly immunogenic antigens such as Ovalbumin (45), CSP (53), and H1N1 (54) produce consistent results in generating a robust B cell response and protective efficacy in preclinical models. MCT^®^ was found to possess high protein-binding capacity (adsorption compatibility with the antigens) (54, 63). In the influenza study, a close correlation of haemagglutination inhibition and neutralization titres in groups formulated with MCT^®^ or alum suggests that the two adjuvants were inducing functionally equivalent influenza-specific Abs. Leuthard et al., 2018 using Ovalbumin, highlighted similar findings (45). However, key differences related to MCT^®^’s physicochemical properties (particulate structure), depot function and biased Th1 specificity highlights some key distinctions of the platform that should be considered when assessing other adjuvants to combined, tailor and optimise the immune response appropriately for specific disease applications.

## MCT^®^ in Virus-Like Particle Formulations: Helping Overcome the Challenges of Modern Vaccines

VLPs can be engineered a specific way to modulate the immune response. In pathogen-specific prophylactic applications, they have proven to be well tolerated and highly immunogenic. The 21^st^ century sees further advancements of the technology harnessing state-of-the-art techniques in leveraging the platform to tackle complex diseases. Mohsen et al., 2020 pay tribute to these advancements in the context of the design, delivery and draining dynamics of VLPs (29) and their respective stages of clinical development and success (30). **Table 3** summarizes immunological mechanisms of VLP-based vaccines in the context of tailoring VLP-platforms with MCT^®^ as an adjuvant system.

**Table 3:** Immunological mechanisms of VLP-based vaccines complement other adjuvants like MCT^®^ and may provide added benefit (29, 43, 45, 47–66–68).

VLP scaffold	MCT^®^
Repetitive and native antigen display - optimal BCR-crosslinking (PAMP; Pathogen Associated Molecular Pattern)	Local inflammation (early innate responses)
Complement activation	Inflammasome activation
Recognition by natural Abs and other innate humoral factors	DC activation
	Particulate for APC targeting
	B cell activation T cell activation
Fast – transient migration to draining lymph nodes	Depot – prolong immune-exposure
Co-delivered adjuvant (e.g., TLR-ligands)	

In regards to targeting B cells and Abs, a major factor here relates to the size and ability of VLPs to display antigens in optimal fashion [repetitive antigen display, Pathogen Associated Structural Patterns (PASPs)], resulting in very robust induction in Ab responses. In an elegant study by Link and colleagues, 2012 the importance of size and repetitive structure as critical factors for efficient Ag presentation to B cells was demonstrated. In this case, IgM Abs which VLPs are recognized by, recruit the complement component C1q followed by activation of C3, resulting in persistent deposition of antigen on follicular dendritic cells (FDCs) *via* complement receptor CD35 (29, 69). Furthermore, the physical association of a repetitive antigen display distanced by 5–10 nm permit optimal B cell receptor crosslinking. The size of VLPs (20–200 nm) enable efficient fast and transient trafficking of native antigen to the lymph nodes highlighting pharmacokinetic advantages of the platform and their ability to target APCs to orchestrate a robust adaptive response (66).

Vaccines targeting pathogens that are more complex will need to induce both B and effector T cells, which is where adjuvant design may come into play more deeply. If our understanding related to the mode-of-action of depot adjuvants/immunomodulators, continue to grow and become more well established, effective rational approaches in VLP vaccine design may be taken in tailoring *dynamic responses* of *desired specificity*.

Adjuvants physically associated with VLPs (e.g., TLR ligands) may enhance B cell responses. Prokaryotic RNA is known to be more effective and superior in this regard and, most importantly, is the ability of this adjuvant-effect to help differentiate a memory B cell pool into secondary plasma cells, which produce very high levels of Abs. This may allow for more efficient and rapid control of an evolving pathogen (67, 70, 71). The CuMV_TT_ VLP is an example of this, based on an ssRNA plant virus, engineered to harbour a universal T cell epitope derived from the tetanus toxoid, to optimise T cell help for B cells (51). The CuMV_TT_ encapsulates pRNA, which acts as a TLR 7/8 ligand. This particular platform has been remarkably effective in generating proof of concept data in different veterinary vaccines for insect-bite hypersensitivity in horses (IL-5), atopic dermatitis in dogs (IL-31), and preclinical PoC in allergy (peanut and cat), pain in osteoarthritis (NGF), Zika virus infection (ED-III), psoriasis (IL-17a), and malaria (PvTRAP and PvCSP) (51–53, 72–78).

### CuMV_TT_ in a Peanut Allergy Model

Where allergic disease is concerned, VLPs have achieved preclinical proof of concept and are subject to further clinical development, notably for peanut allergy (32, 74). Here, targeting B cells using CuMV_TT_ combined with a *single major allergen*, was able to protect against a complex peanut extract in a murine anaphylaxis model (74). In this study mice were immunized with one of three vaccines containing either a mixture of allergens found in whole extract of roasted peanut or with just one single, purified peanut allergen (“Ara h 1” or “Ara h 2”). Regardless of which vaccine was used, immunization strongly reduced systemic and local allergic symptoms in vaccinated subjects and protected against anaphylaxis upon subsequent challenge with a whole peanut allergen mixture. The fact that one injection against a single allergen was sufficient to induce protection against a whole peanut allergen mixture has never been described before and could be applied in different relevant allergies. In addition, the vaccine proved hypoallergenic as previously described (79), which in peanut allergy is a vital characteristic to avoid anaphylactic reactions upon dosing and to improve patient uptake.

### CuMV_TT_-MCT^®^ in a Malaria Disease Model

The inclusion of the depot adjuvant MCT^®^ has highlighted the effectiveness of prolonged physical release of VLP nanoparticles, which have been shown to be particularly effective at priming effector T cell responses. In a number of different comparative adjuvant studies in disease challenge models for Malaria (*P. vivax*) and Cancer (Melanoma), a step-wise improvement in biomarkers/disease progression, with the addition of MCT^®^, has been consistently demonstrated (50, 52, 53). In these studies, the CuMV_TT_ VLPs was screened in a comparative adjuvant study with alum. **Table 4** summarizes the findings from a comparative adjuvant study using CuMV_TT_ in the Malaria disease model, which highlights the effectiveness in combining nanoparticles with MCT^®^ as an optimal way to formulate VLP-vaccines, taking advantage of the physiological properties of the lymphatic system.

**Table 4:** Summary of vaccine efficacy with MCT^®^ and Alum –depot adjuvants. The respective studies conjugated CuMV_TT_ with TRAP or a CSP antigen from *P. vivax* (independent of CuMV_TT_). Formulations were compared against vaccines formulated with Alum.

Protection against *Plasmodium berghei/vivax*					
Formulations screened	Humoral response	Cellular response (CD8+ T cells)	Vaccine efficacy in survival challenge	Reference
**CMVtt-PvTRAP + MCT^®^** ****	** (PvTRAP + MCT^®^) IgG2b > IgG2a > IgG1	** (PvTRAP + MCT^®^)	*** (PvTRAP + MCT^®^)	(52)
**CSP + MCT^®^** ****	** (CSP + MCT^®^) IgG2a > IgG2b > IgG1	N.D	* (CSP + MCT^®^)	(53)

***p = 0.0001 **p = 0.001; *p = 0.01 (one week after second boost); N.D; not determined.

In this study, the vaccine efficacy in the malaria survival challenge models were significantly improved if the vaccines were formulated with MCT^®^, compared to alum. This was explained, in part, due to the high and sustained Ab titres induced in a step-wise improvement by adding MCT^®^ (compared to non-adjuvanted groups) which indicated a more polarized Th1 biomarker specificity compared to alum as indicated by the IgG subset data (see **Table 4**).

### CuMV_TT_-MCT^®^ in a Cancer (melanoma) Model

**Figure 4:**
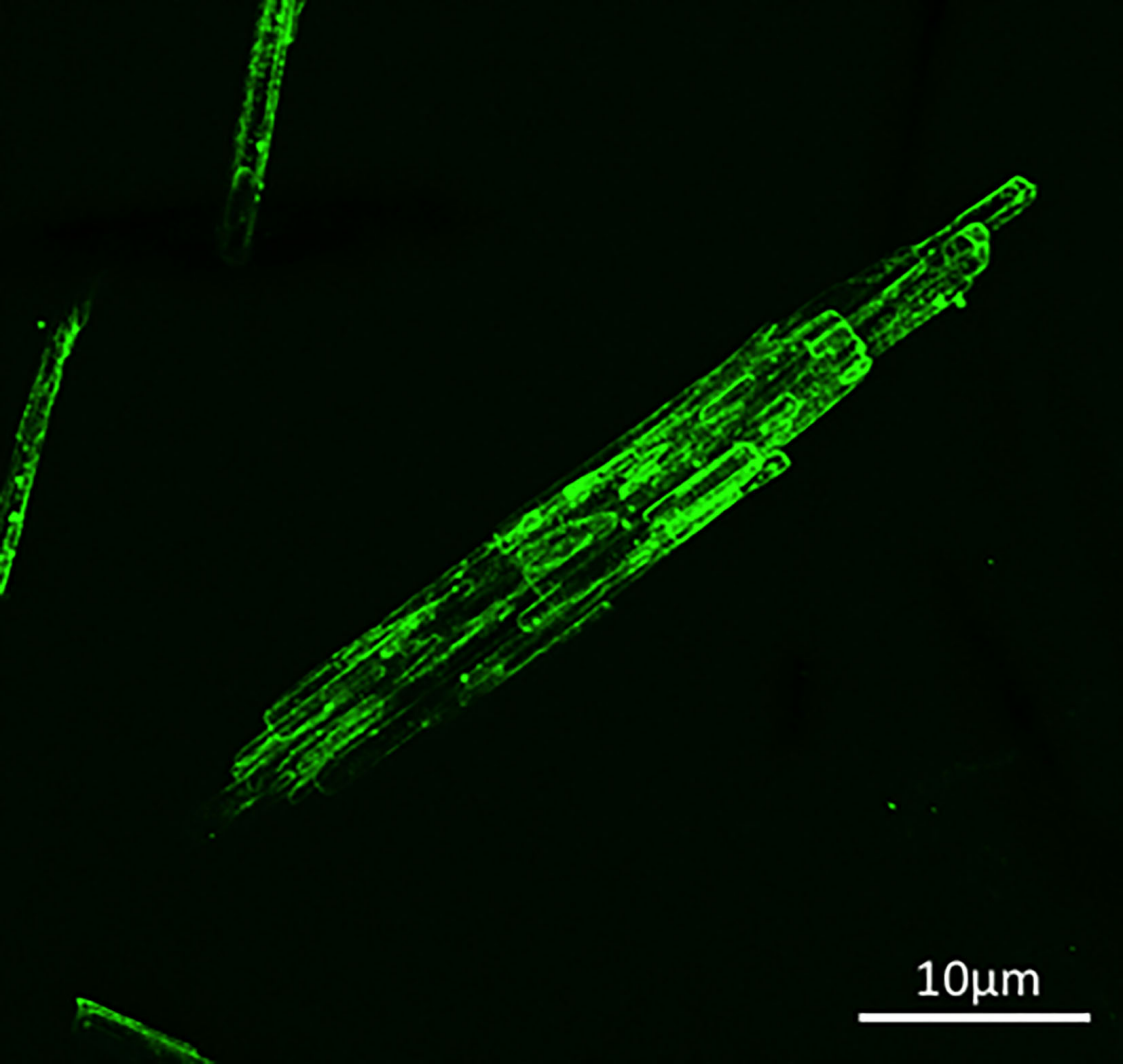
Confocal microscopy imaging of fluorescent dye AF488 CuMV_TT_-p33 nano-vaccine following formulation with the MCT^®^ (20 mg/ml) adjuvant.

Combining CuMVTT_TT_- VLPs displaying T cell epitopes with MCT^®^ as an adjuvant has been tested in an aggressive transplanted melanoma murine model B16F10. The results showed improved anti-tumor efficacy when formulating the nano-vaccine with the micro-sized adjuvant MCT^®^ (**Figure 4**). This hybrid system facilitated an optimal delivery of the vaccine to efficiently prime the adaptive immune system. Furthermore, the MCT^®^ adjuvant was as potent as B type CpGs in a direct comparative assessment of efficacy. These findings highlight the translational potential for application for any solid tumor.

## Conclusions

- MCT^®^ is the crystalline formulation of the non-essential amino acid L-Tyrosine, biodegradable, with an estimated half-life of 48 h at the site of injection (44, 80).
- Formulated as a depot for controlled release from injection site - immunomodulation with allergens, antigens, whole cells, polysaccharides, and lipids.
- Characterized adsorption capacity and stability (broad vaccine scope) facilitating Th1-specific immunological augmentation.
- MCT^®^ and Alum [AlO(OH)] are both distinct crystalline depot adjuvant formulations and induced broadly comparable B- and T-cell responses in mice (45).
- MCT^®^ induced less Th2 polarisation than Alum (less IL-4 and IgE). A higher ratio of IgG/IgE (i.e., relatively higher IgG to IgE) which has been reported to be a surrogate marker indicative of efficacy of AIT in humans (81).
- MCT^®^ facilitates induction of CD8 T-cell responses (45, 50).
- AIT with MCT^®^ adjuvanted allergens induce protection in a mouse model of anaphylaxis (45) and is formulated (adsorbed) with MPL^®^ as an adjuvant system to provide short-course AIT in humans.
- MCT^®^ induces IL-1β secretion *in vitro*, but inflammasome activation does not affect B- and T-cell responses *in vivo* (45).
- MCT^®^ acts independent of TLR activation (45).
- The combination of MCT^®^ with poorly immunogenic antigens such as Ovalbumin (45), CSP (53) and H1N1 (54) produce consistent results in generating a robust B cell response and protective efficacy in preclinical challenge models.
- The adsorption of MCT with CuMV_TT_ virus-like-particles demonstrates significant added benefit in enhancing immunological (B and T cells) responses in Malaria and Cancer (Melanoma) preclinical disease models (50, 52).

## Author Contributions

MH conceived and wrote the manuscript. MB, TK, MK, and MS are senior authors and chief or principal investigators of the MCT-based research and contributed to the scope and discussions. SH and MS are inventors of MCT technology and contributed to the scope and discussion. TC and MM designed the figures. P-JK contributed to the clinical development sections. All authors contributed to the article and approved the submitted version.

## Funding

MM and MB is associated with the following funding agencies, which supported work reviewed in this manuscript; Qatar National Research Fund (PDRA4-0118-18002) and Swiss Cancer League (KFS-4291-08-2017).

## Conflict of Interest

MH, P-JK, MK, TC, SH, and MS are all employees of Allergy Therapeutics Plc (ATLp) who develop and manufacture immunotherapies and diagnostics, including the MCT adjuvant. MM, MB, and TK are under consultancy agreements with ATLp. ML was employed by Bencard Allergie GmbH. MB and TK are co-founders of Saiba GmbH who have out licensed vaccine development of CuMV_TT_ to ATLp within allergy and other disease indications.

The remaining authors declare that the research was conducted in the absence of any commercial or financial relationships that could be construed as a potential conflict of interest.
